# An Asymmetric Microfluidic/Chitosan Device for Sustained Drug Release in Guided Bone Regeneration Applications

**DOI:** 10.3390/bios12100847

**Published:** 2022-10-09

**Authors:** Xin Shi, Beibei Ma, Hongyu Chen, Wei Tan, Shiqing Ma, Guorui Zhu

**Affiliations:** 1 School of Chemical Engineering and Technology, Tianjin University, Tianjin 300350, China; 2 School and Hospital of Stomatology, Tianjin Medical University, Tianjin 300070, China; 3 Department of Stomatology, The Second Hospital of Tianjin Medical University, Tianjin 300211, China

**Keywords:** microfluidic, drug release, guided bone regeneration

## Abstract

One of the major challenges of guided bone regeneration (GBR) is infections caused by pathogen colonization at wound sites. In this paper, an asymmetric microfluidic/chitosan device was developed to release drugs to inhibit infections and to ensure that guided bone regeneration can be realized. The microfluidic technique was introduced into the GBR membrane for the first time, which demonstrated more controllable drug release, more flexible clinical use and had a lower cost compared with surface treatments and embedded nanoparticles. Based on the theory of diffusion and Fick’s first law, the contact area and concentration gradient were adjusted to realize sustained drug release. The standard deviation of minocycline release over 5 days was only 12.7%, which was lower than the joint effect of porous chitosan discs and nanospheres. The in vitro experiments against *E. coli* and Streptococcus mutans showed the excellent antibacterial performance of the device (>95%). The in vitro experiments for fibroblasts at the microfluidic side and osteoblasts at the chitosan side showed the satisfactory biocompatibility and the ability of the device to enhance bone regeneration. Therefore, this microfluidic/chitosan device is a promising therapeutic approach to prevent infection and guide bone regeneration.

## 1. Introduction

The guided bone regeneration (GBR) technique has been commonly performed to repair bone defects caused by pathologic lesions or to augment alveolar bones for dental implant treatment [1]. The core of this technique involves using a barrier membrane to cover the bone defect area, which prevents the migration of epithelial cells and the surrounding fibroblasts from interfering with bone formation. Current GBR membranes fulfill the following five design criteria: (1) biocompatibility; (2) proper stiffness for space maintenance; (3) prevent epithelial cell migration; (4) tissue integration and (5) clinical manageability [2,3,4,5].

In addition to these basic characteristics, GBR membranes are supposed to have some additional functional characteristics. Collagen membranes have advantages such as weak immunogenicity and weak cytotoxicity compared with polytetrafluoroethylene (PTFE) membranes and titanium mesh [6,7]. Bio-Gide^®^ is the leading collagen membrane for oral bone regeneration. The smooth side of this membrane prevents soft tissue from growing into the defect, meanwhile the rough side serves as a framework for bone cells and blood vessels [8,9]. However, collagen membranes usually involve a complex manufacturing process and it is hard to deal with wound infections when using these membranes.

In order to promote antibacterial properties, various GBR membranes were developed [10,11,12]. A common method involves combining some inorganic or organic ingredients with substrates. Chitosan has been recognized as an antimicrobial barrier membrane in GBR and guided tissue regeneration (GTR) [13,14]. Choi et al. showed that chitosan effectively inhibited Actinobacillus actinomycetemcomitans, the representative oral pathogen [15]. Shao et al. [16] fabricated a chitosan/poly(ethylene oxide) membrane with silver nanoparticles to inhibit Porphyromonas gingivalis and Fusobacterium nucleatum. In addition, graphene oxide (GO) can reduce bacteria viability by degrading their cell membranes [17,18]. Zhang et al. [19] reported a multifunctional bilayer GBR membrane using CS, GO, and a type of CaSi (xonotlite) nanowires as a building block. Although this method has advantages in its design and manufacture, the antibacterial properties of the membranes are limited by their intrinsic properties and these are difficult to adjust.

Loading drugs into the membranes directly or via nanoparticles can efficiently inhibit bacterial proliferation [20,21,22]. Chang et al. [23] developed gelatin/hyaluronic acid-based hydrogel membranes loaded with hinokitiol. The membranes were immersed in hinokitiol solution for loading and showed antibacterial properties after 48 h. Lian et al. [24] fabricated a bi-layered GBR membrane, whose loose layer was incorporated with dexamethasone-loaded mesoporous silica nanoparticles (DEX@MSNs), while the dense layer was loaded with the broad-spectrum antibiotic doxycycline hyclate (DCH). In vitro drug release tests indicated that both DEX and DCH followed a favorable sustained release profile. In our previous work [25], we prepared a novel asymmetric microfluidic/chitosan GBR membrane that contained minocycline-loaded nanoparticles. The bacteriostatic rates of this membrane against Porphyromonas gingivalis and Fusobacterium nucleatum were 95.3% and 92.1%, respectively.

Although many drug-loaded membranes have shown their potential, there are still many challenges in manufacturing an ideal antibacterial GBR membrane, such as (1) more precise control of the antibacterial drug release; (2) customized and flexible use in clinical treatments; and (3) low manufacturing cost. For most conventional drug-loaded membranes, the drug is easily burst-released when it is loaded on the surface, while it is difficult to be released when it is loaded inside. In addition, the type and dosage of the drugs are determined along with the manufacture process. Dentists cannot customize the membrane according to the characteristics of the patients. The manufacturing cost should also be considered. Some reported antibacterial GBR membranes need to be manufactured following complex processes, which are only suitable for laboratory scale tests and it is difficult for them to be mass produced.

In recent years, the microfluidic technique has received increasing attention with regard to drug release due to its precise control of fluids and suspended particles [26,27,28]. Meng et al. [29] proposed a micro-high intensity focused ultrasound (MHIFU) generated by a microfluidic device, which is able to control the drug release from temperature-sensitive liposomes (TSL). The flow cytometry results show that the drug delivery under MHIFU sonication leads to a significant increase in apoptosis. Jiang et al. [30] presented a low-cost, passive and flexible microfluidic device that uses wound pH as a trigger for localized drug release. The device can dispense 50 μL onto a 160 mm^2^ dermal coverage within 4 h, showing the precision and controllability of the microfluidic technique. Trani et al. [31] described a subcutaneously implantable remote-controlled nanofluidic device that is capable of sustained drug release with adjustable dosing and timing, demonstrating the advantage of the microfluidic technique in treatment precisely tailored to individual needs.

**Figure 1:**
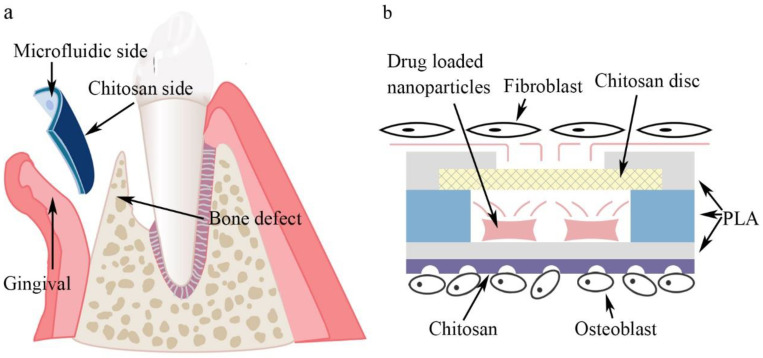
Conceptual illustration of the microfluidic/chitosan device for guided bone regeneration. (**a**) The microfluidic/chitosan device is placed between the gingival region and the bone defect region. (**b**) The working principle of the microfluidic/chitosan device. It can inhibit bacteria proliferation on the microfluidic side and promote bone growth on the chitosan side.

In this study, a novel asymmetric microfluidic/chitosan device was developed for GBR applications. As shown in Figure 1, the microfluidic side prevented fibroblasts from invading bone defects using dense polylactic acid (PLA) substrates (PLA has been approved by European authorities and the Food and Drug Administration for the treatment of periodontal disease and is widely used [32,33,34]), and sustained release of antibacterial drugs from the embedded reservoir at the gingival region was reported. The reservoir can be preloaded with drug-loaded nanoparticles and further filled with customized solutions in clinical use. The chitosan side could facilitate osteoblast adhesion and proliferation at the bone defect region. Minocycline was selected to explore the in vitro drug release profiles under different drug-loaded strategies. The antibacterial performance of the microfluidic/chitosan device was tested against Escherichia coli and Streptococcus mutans. The fibrogenic and osteogenic properties of the microfluidic/chitosan device were further examined using L929 fibroblasts and MC3T3-E1 osteoblasts, respectively. The microfluidic/chitosan device can be manufactured by simple methods using common and commercial materials, which leads to their wider application. The developed microfluidic/chitosan device with both osteogenic and antibacterial properties may be a promising candidate for the GBR technique.

## 2. Materials and Methods

### 2.1. Device Design and Manufacture

**Figure 2:**
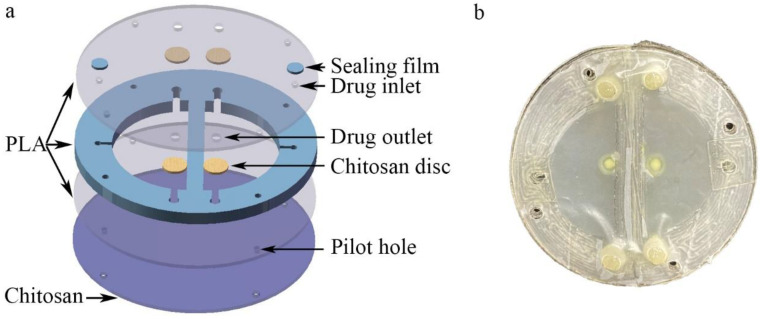
Structure of the microfluidic/chitosan device. (**a**) Three-dimensional exploded view of the device, from top to bottom: top PLA layer, drug reservoir, bottom PLA layer, and chitosan layer. (**b**) Photograph of the microfluidic/chitosan device.

The microfluidic/chitosan device shown in Figure 1a consisted of three PLA layers, porous chitosan discs, a sealing film and a chitosan layer. The top and the bottom PLA layers were cut from the PLA film (Hongjian Bio-medical Products Co. Ltd., Guangzhou, China) using a laser (GMA6040, Yueming Laser CO., Dongguan, China). The cutting speed was 250 mm/s. The middle PLA layer was 800 μm and constructed using a fused deposition modeling (FDM) 3D printer (JGAURORA A6, JGMAKER CO., Shenzhen, China). The structure of the middle PLA layer was designed with AutoCAD 2018 and then transferred to the 3D printer. The printing temperature was 210 °C and the printing velocity was 80 mm/s. The XY printing accuracy was 0.05 mm and the minimum Z plane resolution was 0.1 mm. The cavity within the three PLA layers was the drug reservoir, whose volume was 265.38 mm^3^. The porous chitosan discs were placed between the top PLA layer and the middle PLA layer. For chitosan layer synthesis, 2% chitosan solution was prepared using 2 g of chitosan powder and 100 mL of 2% ice acetic acid and left overnight at 4 °C to remove the bubbles. Then, the chitosan solution was slowly poured into a polytetrafluoroethylene mold (60 mm in diameter, 15 mm in depth) to obtain a homogeneous film. The films were then immersed into liquid nitrogen for 10 s. Subsequently, these chitosan films were lyophilized at −80 °C for 24 h to produce a porous structure. The inlets were used to inject the drug solution. After injection, the inlets were blocked by a sealing film. The structure of the microfluidic/chitosan device is shown in Figure 2. All components are bonded by tissue adhesive. The total thickness of the device was about 2 mm (50 μm for the top and bottom PLA layer, 800 μm for the middle PLA layer and about 1 mm for the chitosan layer). The area of the device can be adjusted according to the bone defect area and the internal structure can be scaled proportionally. To facilitate testing, the radius of the device in the in vitro experiments was 10.2 mm. When placed into the oral position, the radius of the device can be reduced to 2–5 mm.

### 2.2. Drug Release Characterizations

To characterize the drug release capability, the microfluidic/chitosan devices loaded with minocycline solution and nanoparticles (PERIOCLINE, Sunstar INC., Osaka, Japan) were placed in beakers with 10 mL PBS (pH 7.4) and gently shaken at a constant temperature of 37 °C. Subsequently, the supernatant was collected at various time points and inspected by a liquid chromatograph mass spectrometer (Waters E2695). The minocycline concentration was calculated based on the standard curve and used to calculate cumulative release [35]. All calculations were repeated more than three times and averaged.

### 2.3. In-Vitro Assessment of Bacterial Infections

The proliferation of the Escherichia coli and Streptococcus mutans were evaluated to measure the antibacterial performance by measuring the OD value [36]. The bacteria were seeded in 96-well plates (1 × 10^7^ colony forming units (CFU)/mL) using the leachate of the microfluidic/chitosan device). Then, 10 μL of bacterial suspension was used to obtain the OD value at particular timepoints. The morphologies of the top PLA layer and the bacteria were observed by scanning electron microscopy (SEM; Nova NanoSEM 430, FEI, Hillsboro, OR, USA).

### 2.4. In-Vitro Assessment on Fibroblast Cell Proliferation

The proliferation of the fibroblast L929 on the microfluidic side was evaluated using the normal CCK-8 assay [37]. The fibroblasts in different conditions were seeded in 96-well plates (2000 cells/well). Then, 10 μL of CCK-8 reagent (Solarbio, Beijing, China) was added to the parallel wells every 24 h, followed by incubation for 4 h at 37 °C. The OD value was calculated at 450 nm absorbance. Live/dead cell double staining (Solarbio, Beijing, China) was used to distinguish dead cells (red staining) from live ones (green staining) to evaluate the viability of fibroblasts and the biocompatibility of the microfluidic/chitosan device. The fibroblasts were seeded on the device in 24-well plates (40,000 cells/well). Then, the cells were washed with PBS three times, and then stained with AO/EB (acridine orange/ethidium bromide) solution (0.5 mL/well) at various timepoints. The media were refreshed every 24 h. The fibroblasts were observed using laser confocal microscopy (CMLS) (Fv-1000, Olympus, Tokyo, Japan). The morphology of the fibroblasts on the top PLA layer surface was observed by SEM. All tests were repeated at least 3 times.

### 2.5. In-Vitro Assessment of Osteoblast Cell Proliferation

The proliferation and the viability of the osteoblasts MC3T3-E1 on the chitosan side were also evaluated by using the CCK-8 assay. The method was the same as the method mentioned in Section 2.4. The morphology of the osteoblast on the chitosan device surface was observed by SEM.

## 3. Results and Discussion

### 3.1. Mechanism of Drug Delivery at the Microfluidic Side

The antimicrobial effect of the microfluidic/chitosan device is based on the imbedded drug. When the microfluidic/chitosan device was immersed into culture medium in vitro (or pasted to gum tissue in vivo), minocycline was released due to molecular diffusion. The release amount *Q* is determined as follows:

(1) Q=JAt

where *J* is the diffusion flux, *A* is the contact area and *t* is the time. The diffusion flux, *J*, can be calculated as Fick’s first law, which is as follows:

(2) $$J=-D\frac{\mathrm{dC}}{\mathrm{dx}}$$

where *D* is the diffusion coefficient, *C* is the concentration of the solute and *x* is the position. Thus, $\frac{\mathrm{dC}}{\mathrm{dx}}$ is the concentration gradient. It shows that the solute will move from a region of high concentration to a region of low concentration. The diffusion coefficient, *D*, can be estimated by the Wilke–Chang formula, which is as follows:

(3) $$D=7.4\times 10^{-8}\left[{\left(\varphi \mu_{B}\right)}^{1/2}\frac{T}{\eta_{B}V_{A}^{0.6}}\right]$$

where *φ* is the parameter of association of the solvent, *μ*_B_ is the molecular mass of the solvent B, *V*_A_ is the molar volume of solute A at its boiling point under normal conditions, *η*_B_ is the substance viscosity and *T* is the temperature. According to the formulas above, for a certain drug solution at a certain period and temperature, the amount of drug released depends on the contact area and drug concentration gradient. In this microfluidic/chitosan device, the contact area is controlled by the porosities of chitosan discs. The drug concentration gradient can be adjusted flexibly by drug dosage and the proportion of solution and nanoparticles.

### 3.2. Minocycline Release Performance

**Figure 3:**
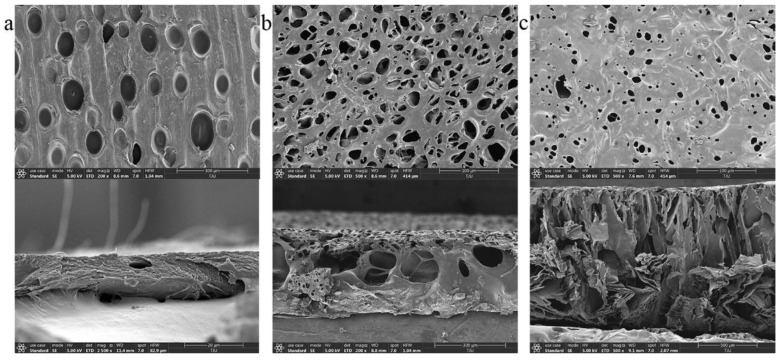
Images of chitosan discs using a scanning electron microscope (SEM). (**a**) SEM image of chitosan disc A, left for 2 h for dry phase separation. (**b**) SEM image of chitosan disc B, left for 1 h for dry phase separation. (**c**) SEM image of chitosan disc C, left for 0.5 h for dry phase separation.

In order to control the release velocity, chitosan films with various porosities were synthesized and cut into discs. Chitosan solution was slowly poured into a polytetrafluoroethylene mold at room temperature to obtain an even liquid film. Then, the liquid film was pre-heated at 45 °C for 2 h (Figure 3a)/1 h (Figure 3b)/0.5 h (Figure 3c) for dry phase separation, before immersion into the sodium hydroxide solution for wet phase separation. Subsequently, the obtained chitosan film was washed repeatedly with the distilled water until the pH value was neutral. Based on different operation parameters, three types of chitosan films were synthesized and punched into discs, whose diameters were 1.5 mm. As shown in Figure 3, the size and total area of the pores on disc C’s surface were smaller than that on A and B. In addition, the internal structure of the disc became more complicated as the pore size decreased. In summary, the disc C can minimize the contact area between the embedded reservoir and external PBS.

**Figure 4:**
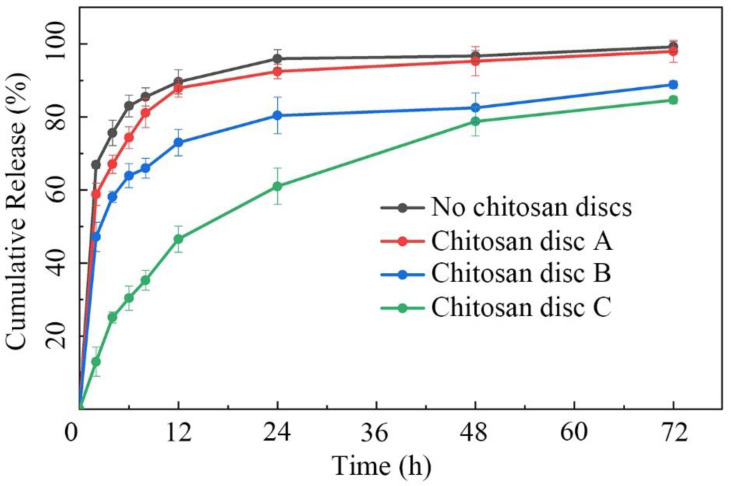
Cumulative release plot of minocycline in 72 h. The minocycline burst-released within 12 h in the microfluidic/chitosan device with no discs/disc A/disc B. The minocycline demonstrated sustained release over 48 h in the microfluidic/chitosan device with disc C. Error bars represent the standard deviation of the mean.

The three types of chitosan discs were then placed into microfluidic/chitosan devices. After injected with the same volume of minocycline solution (0.25 mg/mL), the devices were submerged into 10 mL PBS (pH 7.4). The cumulative release curves of different devices were calculated and are shown in Figure 4. For the control with no chitosan discs, the contact area was the whole drug outlet. The mass transfer efficiency reached the theoretical maximum of this device. The total release was defined as 100% at 72 h. About 90% of minocycline was released over 12 h. For the device with disc A, the release velocity was similar to the control. The large holes induced an insufficient decrease in the contact area. Thus, minocycline still burst-released within 12 h. For the device with disc B, the 12 h release and 72 h release of minocycline were decreased to 73% and 88%, respectively. The decrease in the 72 h release may be caused by a higher dead volume in the internal caves, which were connected to the drug reservoir. About 12% minocycline remained in the device and was not easily released. Thus, the relative release rate, the ratio of 12 h release to 72 h release, is a more accurate parameter to measure sustained-release performance. The relative release rates of devices with disc A and disc B were calculated as 89.9% and 83.0%, respectively. It indicated that disc B had an indistinctive effect on reducing the mass transfer rate. For the device with disc C, the 12 h release and the 72 h release rates were 54.9% and 84.6%, respectively. The 72 h release showed that the dead volume of disc C was similar to that of disc B. The 12 h release showed that the disc had the capability to control the mass transfer rate by adjusting the contact area with limited drug retention. Thus, the microfluidic/chitosan device with disc C was selected for further tests. The original data of this experiment from the liquid chromatograph mass spectrometer are presented in Table S1 in the Supplementary Materials.

**Figure 5:**
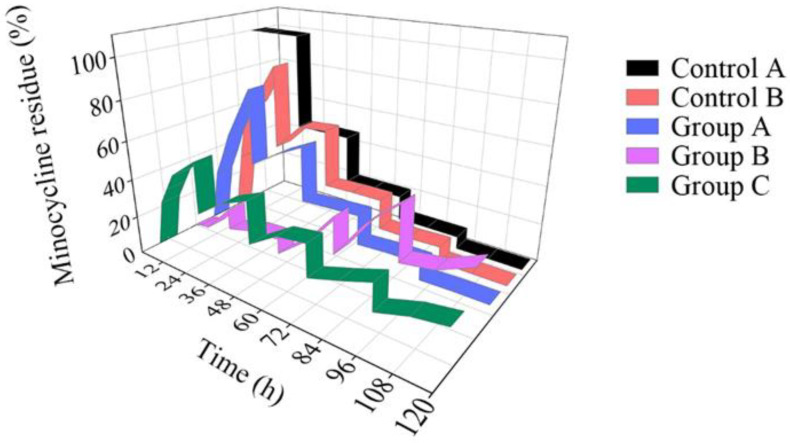
Minocycline residue over 120 h. For the solution (black belt) in the dish, the minocycline residue decreased rapidly. For the nanoparticles (red belt) in the dish and the solution in the microfluidic/chitosan device (blue belt), the amount of minocycline residue in the initial period was much higher than that in the terminal period. For the nanoparticles in the microfluidic/chitosan device (purple belt), the amount of minocycline residue in the initial period was much lower than that in the terminal period. When a mixture of the solution and the nanoparticles was loaded into the microfluidic/chitosan device (green belt), the minocycline residue was stable throughout the whole period.

Besides contact area, the mass transfer was also affected by the minocycline concentration gradient. To simulate the metabolism of gum tissue, we refreshed 50% of the PBS (5 mL) every 24 h. Minocycline residue in the dish before and after refresh was acquired and standardized. As shown in Figure 5, when the minocycline solution (0.25 mg/mL) was directly added to the dish without the microfluidic/chitosan device (control A, black belt), the amount of minocycline residue was halved with each PBS refresh. On the other hand, the amount of minocycline residue quickly increases due to the gradient shock caused by the first PBS refresh when the minocycline solution (0.25 mg/mL) was added into the device (Group A, blue belt). In the period of 24 h to 48 h, the minocycline release rate decreased steeply due to insufficient minocycline reserves. After 4 refreshes, the 120 h minocycline residue was only 8.9%. The standard deviation of minocycline residue at different time points was calculated to quantify the release fluctuation. For the solution in the dish and solution in the microfluidic/chitosan device, the SD values were 38.1% and 32.8%, respectively. The high SD value indicated that minocycline did not demonstrate sustained release throughout the whole test period. To overcome the burst-release in the initial 48 h, nanoparticles loaded with minocycline were selected (Periocline, Sunstar INC). The release character of the nanoparticles (loaded with 80 μg minocycline) in the dish was referred to as Control B, the red belt. Although it decreased the release rate in the early stage, the dramatic decline in minocycline residue over 120 h led to a large SD of 29.2%. Then, we injected a mixture of PBS and nanoparticles (loaded with 80 μg minocycline) into the microfluidic/chitosan device. Confined by the nanoparticle and chitosan disc, it was difficult for minocycline molecules to diffuse to the external environment in the initial 48 h. The residue did not reach its peak until 96 h (Group B, purple belt). The SD decrease to 14.9% reflected a more stable release process. However, in order to avoid surgical site infection (SSI), the local minocycline concentration should increase to the same value as the bacteriostatic concentration as soon as possible. This means that the characteristics of Group B do not match the clinical requirements. Finally, a mixture of minocycline solution (0.25 mg/mL) and nanoparticles (loaded with 80 μg minocycline) was injected into the microfluidic/chitosan device (Group C, green belt). In the initial 48 h, the minocycline concentration rapidly increased to the same value as the bacteriostatic concentration. The release in the initial period was dominated by the minocycline solution, compared to Group B. From 48 h to 120 h, the minocycline residue increased after each PBS refresh, which is different from the rapid decrease in Group A. It can be deduced that the release in this period was dominated by minocycline-loaded nanoparticles. At the same time, the SD was only 12.7%, which indicated a stable drug release with less flush. In conclusion, the joint loaded method of minocycline solution and nanoparticles in the microfluidic/chitosan device showed its advantages of timeliness and stationarity. The original data of this experiment from the liquid chromatograph mass spectrometer are presented in Table S2 in the Supplementary Information.

### 3.3. Antibacterial Performance of the Microfluidic/Chitosan Device

The antibacterial efficiency of the microfluidic/chitosan device for GBR treatment was evaluated by investigating the optical density (OD) values of the tested bacteria. *Escherichia coli*, the most common strain, and *Streptococcus mutans*, which exists only in the oral environment, were selected for the bacteriostatic tests. The concentration of minocycline solution in the microfluidic/chitosan device was 0.25 mg/mL. The mass of the minocycline loaded by the nanoparticles was 80 μg.

**Figure 6:**
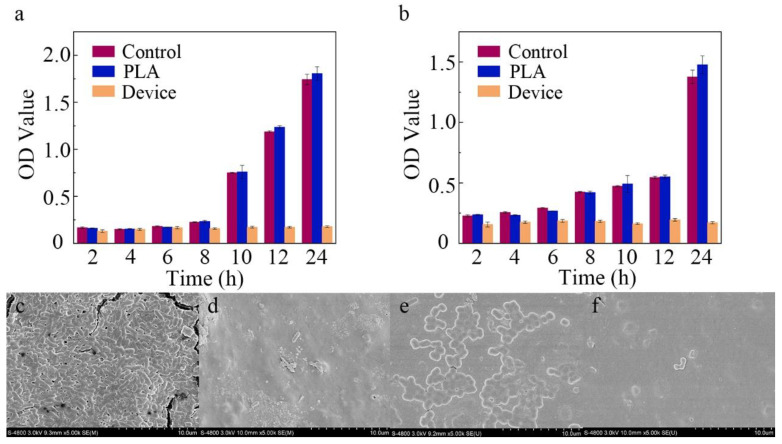
Characterization of antibacterial activity. (**a**) The OD value of *E. coli* after 24 h. (**b**) The OD value of *S. mutans* after 24 h. (**c**) SEM image of PLA substrate surface with *E. coli*. (**d**) SEM image of microfluidic/chitosan device surface with *E. coli*. (**e**) SEM image of PLA substrate surface with *S. mutans*. (**f**) SEM image of microfluidic/chitosan device surface with *S. mutans*.

As shown in Figure 6a,b, after the cultivation of these bacteria with the PLA substrate (blue stripe) for 24 h, the OD values were similar to those of the blank controls (red stripe). As expected from reports [38,39], the PLA substrate had limited influence on antibacterial activity in our experiments. When minocycline was introduced, the proliferation rate of *E. coli* was inhibited by 98.1% ($(1\;-\;\frac{\text{proliferation}\;\text{rate}\;\text{of}\;\text{device}}{\text{proliferation}\;\text{rate}\;\text{of}\;\text{control}})\;\times \;100\%$). The SEM images of surfaces of the PLA substrate and microfluidic/chitosan device are shown in Figure 6c and Figure 6d, respectively. For *S. mutans*, the 24 h inhibition rate reached 96.0% in the presence of the microfluidic/chitosan device. The SEM images of surfaces of the PLA substrate and microfluidic/chitosan device are shown in Figure 6e and Figure 6f, respectively. These results indicated that the bacteriostasis of the microfluidic/chitosan device can be mainly attributed to minocycline. The antibacterial mechanisms of minocycline include inhibiting protein synthesis by binding at the decoding center of the small subunit [40,41].

### 3.4. Fibrogenic Performance of the Microfluidic/Chitosan Device

**Figure 7:**
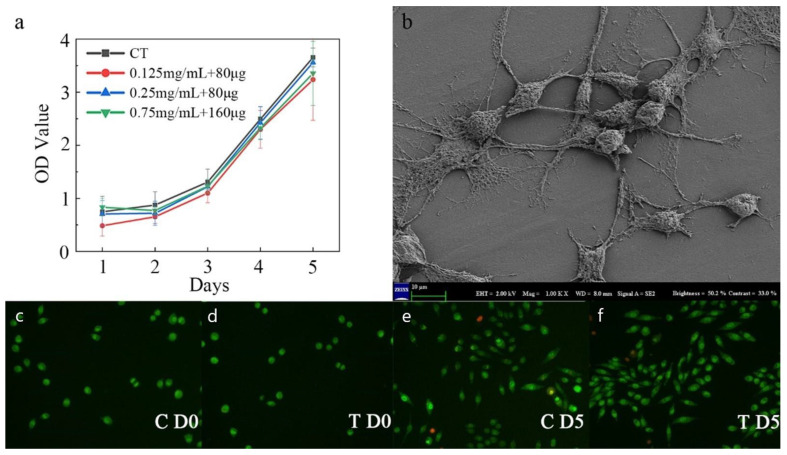
Characterization of fibrogenic performance. (**a**) The OD value of the control and minocycline conditions across 5 days. (**b**) SEM image of PLA substrate surface with fibroblast. (**c**) Live/dead staining for control on day 0. (**d**) Live/dead staining for minocycline condition on day 0. (**e**) Live/dead staining for control on day 5. (**f**) Live/dead staining for minocycline condition on day 5.

Figure 7a shows the CCK8 assay results of the fibroblasts seeded on the PLA surface of the microfluidic/chitosan device after 1–5 days. With the same initial seeding density, the OD values of the control and test groups were on a similar level on day 5. The blue line represents the minocycline condition that was same as the condition used in the bacterial test. Under this loading condition, bacterial proliferation was inhibited while the fibroblasts grew normally, which demonstrated the antibacterial and non-cytotoxic feature of the device. The red and green curves represent another two minocycline conditions with lower (0.125 mg/mL for solution and 80 μg for nanoparticles) and higher (0.75 mg/mL for solution and 160 μg for nanoparticles) minocycline loads, respectively. These similar proliferation curves showed a wide range for minocycline load and flexibility in the use of our device. As shown in the SEM results, the dense PLA layer could facilitate cell attachment (Figure 7b). The results of live/dead cell staining using the AO/EB kit are shown in Figure 7c–f. Both cells in the control and minocycline condition (0.25 mg/mL for solution and 80 μg for nanoparticles) spread normally over 5 days and demonstrated spindle morphology.

### 3.5. Osteogenic Performance of the Microfluidic/Chitosan Device

**Figure 8:**
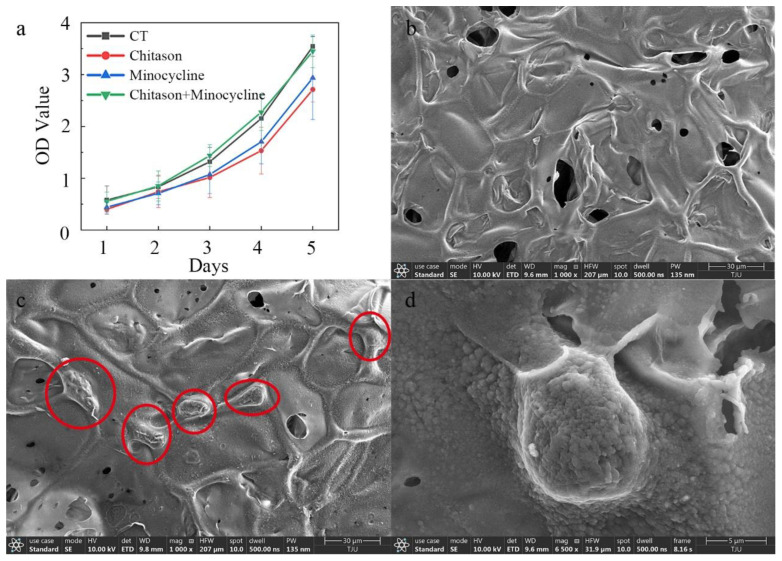
Characterization of osteogenic performance. (**a**) The OD value of the control and minocycline conditions across 5 days. (**b**) SEM image of chitosan substrate surface without osteoblasts. (**c**) SEM image of chitosan substrate surface with osteoblasts (30 μm scale). (**d**) SEM image of chitosan substrate surface with osteoblasts (5 μm scale).

Figure 8a shows the CCK8 assay results of the osteoblasts seeded on the chitosan surface of the microfluidic/chitosan device after 1–5 days. The cells rapidly proliferated and presented an increasing trend, suggesting that the chitosan layer possessed good biocompatibility (red curve). The blue curve and green curve represent the minocycline conditions (0.25 mg/mL for solution and 80 μg for nanoparticles), which suggests that this loading condition was also acceptable for osteoblasts. SEM micrographs of the chitosan surface without osteoblasts are shown in Figure 8b. Based on our previous study, the loose and porous morphology can promote osteoblast adhesion. SEM micrographs of the chitosan surface with osteoblasts (red circles) are shown in Figure 8c. After 3 days of seeding, the osteoblasts showed a round shape and were anchored to the chitosan surface by discrete filopodia, as shown in Figure 8d, suggesting that the device had satisfactory cytocompatibility.

## 4. Conclusions

The novel asymmetric antibacterial microfluidic/chitosan GBR device can be manufactured by PLA layer preparation, chitosan layer synthesis and further assembly. Porous chitosan discs were used to adjust the contact area between the embedded reservoir and external environment. The 12 h release and the 72 h release of the test group decreased to 54.9% and 84.6%, compared to the control group. When injecting a mixture of minocycline solution and minocycline-loaded nanoparticles into the reservoir, the standard deviation of minocycline release was only 12.7% after 5 days, which showed demonstrated sustained release. The 24 h inhibition rate of *E. coli* and *S. mutan* reached 98.1% and 96.0%, respectively. Both fibroblasts at the microfluidic side and osteoblasts at the chitosan side were able to proliferate normally and showed excellent fibrogenic and osteogenic performance. In conclusion, this microfluidic/chitosan device can efficiently prevent infection and the introduction of microfluidics into the GBR technique show potential regarding clinical requirements.

## Data Availability

Not applicable.

## Supplementary Materials

The following supporting information can be downloaded at: https://www.mdpi.com/article/10.3390/bios12100847/s1, Table S1: The minocycline concentration from liquid chromatography-mass spectrometry; Table S2: The minocycline concentration from liquid chromatography-mass spectrometry.
