# Supplementary material for: An Asymmetric Microfluidic/Chitosan Device for Sustained Drug Release in Guided Bone Regeneration Applications
Source: Biosensors (Basel). 2022 Oct 9;12(10):847. doi: 10.3390/bios12100847 (PMC9599337; doi:10.3390/bios12100847)
Supplement: Supplementary file 1 [file biosensors-12-00847-s001.zip › biosensors-1912211-supplementary.pdf]

Supplementary Information

An Asymmetric Microfluidic/Chitosan Device for Sustained Drug Release in Guided Bone Regeneration Applications

Xin Shi [1], Beibei Ma [2], Hongyu Chen [1], Wei Tan [1], Shiqing Ma [3,]* and Guorui Zhu[1,]*

Xin Shi[1], Beibei Ma[3], Hongyu Chen[1], Wei Tan[1], Shiqing Ma[2,]* and Guorui Zhu[1,]*

[1] School of Chemical Engineering and Technology, Tianjin University, Tianjin 300350, China

[2] School and Hospital of Stomatology, Tianjin Medical University, Tianjin 300070, China

[3] Department of Stomatology, The Second Hospital of Tianjin Medical University, Tianjin 300211, China

Table S1. The minocycline concentration from liquid chromatography-mass spectrometry in Figure 4.

Time point (h)	No discs (μg/mL)	Disc A (μg/mL)	Disc B (μg/mL)	Disc C (μg/mL)
0	0	0	0	0
2	4.6839	4.1162	3.3023	0.9114
4	5.2952	4.6949	4.0692	1.7581
6	5.8104	5.2052	4.4757	2.1292
8	5.9854	5.6779	4.6169	2.4712
12	6.2731	6.1528	5.1097	3.2586
24	6.7155	6.4709	5.6284	4.2711
48	6.7673	6.6676	5.7765	5.5138
72	6.9428	6.8567	6.2177	5.9248

Table S2. The minocycline concentration from liquid chromatography-mass spectrometry in Figure 5.

Time point (h)	Control B (μg/mL)	Group A (μg/mL)	Group B (μg/mL)	Group C (μg/mL)
4	2.1822	1.9796	0.2793	1.7433
8	4.1481	2.7825	0.4097	2.3695
12	4.4172	3.6691	0.4337	2.9849
24	6.043	5.5973	1.4822	3.7445
36	4.0138	3.4414	1.0073	2.5828
48	4.0955	3.9584	1.0754	3.011
60	2.0899	2.0494	1.3641	2.1667
72	2.1014	2.1116	2.6249	2.3216
84	1.0453	1.1363	2.7491	1.5156
96	1.0976	1.1619	3.7088	1.9054
108	0.5412	0.6212	2.0396	1.3163
120	0.5436	0.6295	2.5543	1.5087
